# Combined Modality Therapies for High-Risk Prostate Cancer: Narrative Review of Current Understanding and New Directions

**DOI:** 10.3389/fonc.2019.01273

**Published:** 2019-11-29

**Authors:** Benjamin A. Greenberger, Victor E. Chen, Robert B. Den

**Affiliations:** Department of Radiation Oncology, Sidney Kimmel Medical College and Cancer Center, Thomas Jefferson University, Philadelphia, PA, United States

**Keywords:** prostate neoplasms, high-risk, clinically localized, prostatectomy, radiotherapy

## Abstract

Despite the many prospective randomized trials that have been available in the past decade regarding the optimization of radiation, hormonal, and surgical therapies for high-risk prostate cancer (PCa), many questions remain. There is currently a lack of level I evidence regarding the relative efficacy of radical prostatectomy (RP) followed by adjuvant radiation compared to radiation therapy (RT) combined with androgen deprivation therapy (ADT) for high-risk PCa. Current retrospective series have also described an improvement in biochemical outcomes and PCa-specific mortality through the use of augmented radiation strategies incorporating brachytherapy. The relative efficacy of modern augmented RT compared to RP is still incompletely understood. We present a narrative review regarding recent advances in understanding regarding comparisons of overall and PCa-specific mortality measures among patients with high-risk PCa treated with either an RP/adjuvant RT or an RT/ADT approach. We give special consideration to recent trends toward the assembly of multi-institutional series targeted at providing high-quality data to minimize the effects of residual confounding. We also provide a narrative review of recent studies examining brachytherapy boost and systemic therapies, as well as an overview of currently planned and ongoing studies that will further elucidate strategies for treatment optimization over the next decade.

## Introduction

Of the cases of newly diagnosed localized prostate cancer (PCa), ~15% are discovered as high-risk disease (1). There has been clinical equipoise surrounding the issue of selecting optimal definitive therapy, as treatment paradigms have evolved to incorporate both upfront surgery and radiation approaches (2). Definitive therapy for newly diagnosed cases of high-risk disease now routinely includes radical prostatectomy (RP) followed by a consideration of adjuvant radiation (ART) and androgen deprivation therapy (ADT) or a combination of external beam radiation therapy (XRT) with androgen deprivation therapy (ADT) with or without the addition of brachytherapy (BT).

While treatment of favorable-risk localized disease has benefited from the relatively recent publication of randomized controlled data demonstrating no detectable difference in PCa-specific mortality (PCM) between RP- and RT-based approaches (3), there has not been a large-scale randomized clinical trial representing patients with high-risk disease. The available trials in the setting of high-risk PCa that compare outcomes between RP and RT cohorts are limited by small numbers of high-risk patients (4, 5). Comparing RT and RP over the past several decades has been somewhat of a moving target, as paradigms for treatment of high-risk disease have shifted with steady innovation. Recent practice-shaping trends in RT clinical trials and retrospective investigation have explored optimization of combined modality therapies utilizing radiation, including XRT + BT boost (6), demonstration of efficacy and duration optimization of ADT with and without dose-escalated RT (7–15), incorporation of whole-pelvis XRT for high-risk patients (16–18), assessment of safety of hypofractionation in the setting of high-risk disease (19–22), and exploration of addition of systemic therapies (23–26). Despite the rapid advancement of understanding regarding the optimization of modern therapies, the decision regarding primary intervention with RP vs. RT has remained most elusive. As such, we have sought to provide a narrative review focusing on advancements in the understanding of treatment efficacy of optimized RT- and RP-based approaches to clinically localized high-risk PCa. We have also sought to provide a brief overview of upcoming clinical trials anticipated over the next decade.

## Methods

We aimed to review the available literature regarding the relative efficacy of modern strategies incorporating RP or RT-first techniques targeted at the definitive treatment of high-risk PCa. We constructed search terms corresponding to three separate reference databases: PubMed, Scopus, and Cochrane Central Register of Controlled Trials. Subject headings and MeSH terms were incorporated with text/keyword terms for “radical prostatectomy,” “radiotherapy,” “outcome,” “survival,” “mortality, “systemic therapy,” and related terms. These were assembled into search strings tailored for each database (Supplementary Table 1). Given the heterogeneity in classification schemes of PCa (2), we did not discriminate with regard to the definition of high-risk and aimed to target the common definitions (Supplementary Table 2), as well as cohorts constructed to examine subsets of common definitions of high-risk disease, such as Gleason 9–10. Cohorts including, though not exclusively focusing on, locally advanced patients with nodal disease were included, as many investigators did not know nodal status preoperatively. Additional details of the literature search are described in Supplementary Table 1. Given changes in practice patterns concerning RT dose-escalation and ADT use in high-risk disease, we drew principally from studies published within the past decade (since 2009). Reference lists of reviews published on clinically localized PCa were also checked for additional relevant publications (26–32). Our primary outcomes of interest were PCM and overall mortality (OM), although a meta-analysis focusing on these outcomes was not the purpose of this review. We excluded studies focusing primarily on surrogate measures of progression or PCM, such as biochemical recurrence/failure (BCR/BF). Studies focusing on toxicity, patient-reported outcomes, and quality of life are beyond the scope of this review.

The United States national online registry of clinical trials located at clinicaltrials.gov, as managed by the National Library of Medicine at the National Institutes of Health, was queried for all current active trials with keyword terms “prostate cancer,” “prostate cancer Stage III,” and related synonymous terms (Supplementary Table 3). The search was limited to exclude trials that had been suspended, terminated, completed, withdrawn, or trials with unknown status. Each clinical trial in the resulting list was individually reviewed for relevance and inclusion in the table of current trials of interest. Furthermore, multiple large, geographically disparate U.S. academic institutions with searchable lists of active clinical trials including University of California San Diego, University of California San Francisco, Thomas Jefferson University, MD Anderson, and Memorial Sloan Kettering Cancer Center were queried for trials investigating PCa therapies to ensure completeness of the initial clinicaltrials.gov search. Ongoing trials that had been previously quoted or otherwise referenced in the other sources surveyed in this review paper were included in **Table 5**.

### Lack of Data From Randomized Clinical Trials

Randomized controlled trials (RCTs) providing insight into relative efficacy of upfront RP or RT modalities have remained limited for the past several decades, which has prompted the use of alternative comparison methods to address investigation (Supplementary Table 4). There were two early RCTs in PCa, including one performed by the Uro-Oncology research group before routine use of prostate-specific antigen (PSA) and one by the Japanese Study Group for Locally Advanced Prostate Cancer (5, 33). Only the latter provided representation of high-risk disease. Multiple additional studies were initiated and failed to accrue enough patients to investigate mortality endpoints (34–37). These early studies, when reported, demonstrated possibly improved survival outcomes in favor of surgery across localized disease risk groups. However, there were significant concerns regarding stage migration, small sample size, short follow-up, and other methodological limitations of these trials that limited their impact (38). A more recent study of patients with localized or locally advanced PCa undergoing RP or XRT + BT with ADT did not demonstrate a statistically significant difference in OM or PCM. The study was underpowered to assess survival outcomes, though, with only 89 patients (4) (Table 1). Although not providing information regarding high-risk disease, the ProtecT study represents a more modern, well-designed, randomized trial in PCa. The comparison of RT- and RP-based modalities contained in this trial did not demonstrate statistically significant differences in PCM (*p* = 0.48), with OM rates also demonstrably comparable between the arms. Interpretation of this trial concerning its RT vs. RP outcomes is limited due to a lack of statistical power, as the observed PCM was lower than anticipated (38, 44). In the setting of high-risk disease, a modern clinical trial targeting a randomization between upfront RT- and RP-based definitive treatment has recently been initiated with the SPCG-15 trial. This trial compares standard (RT + ADT) and experimental (RP with extended pelvic lymph node dissection and with addition of adjuvant/salvage RT and ADT) treatment at 23 centers in Denmark, Finland, Norway, and Sweden (45) (Table 5). As such an effort is just getting underway, it is likely that for the next decade, conclusions regarding the relative efficacy of RP- vs. RT-based approaches for high-risk disease will not be drawn from randomized data.

**Table 1:** Selected representative institutional studies of comparative effectiveness of radiotherapy and radical prostatectomy in high-risk prostate cancer.

**Study type**	**Representative study**	**Summary description**	**Endpoints**	**Findings related to PCM, OM**	**Key limitations**
Randomized controlled trial	Lennernäs et al. (4) (accrual 1996–2001)	89 patients, T1b–T3a, N0, M0 and PSA ≤ 50 ng/ml. All underwent total androgen blockade (6 months). RP vs. XRT + BT.	Self-reported HRQoL. Secondary endpoints: OM, PCM	10-year results RP−13.3% PCM, 26.7% OM XRT + BT: 4.5% PCM, 20.5% OM No statistically significant differences	Limited sample size, lack of statistical power
Single or limited multi-institutional observational study	Zelefsky et al. (39) Memorial Sloan Kettering (accrual 1993–2002)	2,380 pts (including 409 NCCN high-risk) with T1c-T3b PCa were treated with intensity-modulated XRT (≥81 Gy) or RP	Primary endpoint: distant metastasis. Secondary endpoint: PCM	5-year results with 95% CI RP: 1.0% (0.1–7.0%) PCM RT: 3.7% (1.8–7.4%) PCM	Hazard ratios not reported for high-risk subset. 3–6 months ADT in 56% of patients. No adjuvant ADT in high-risk patients
	Boorjian et al. (40) Mayo Clinic, Fox Chase (accrual 1988–2004)	1,847 NCCN high-risk patients, treated with RP or XRT with pelvic nodes included	Systemic progression, PCM, OM	10-year PCM 8% (RP), 8% (XRT + ADT), and 12% (XRT alone). Worse HR (1.6) for OM for XRT/ADT compared with RP, though not significant for PCM	56% ADT utilization in XRT cohort, low radiation dose of median 72 Gy XRT
	Ciezki et al. (41) Cleveland Clinic (accrual 1996–2012)	2,557 NCCN high-risk patients, treated with RP or XRT (≥78Gy) or BT (LDR 144 Gy)	PCM, BF, clinical relapse	5-year results PCM was 5.3% XRT, 3.2% LDR, and 2.8% for RP	> 6-months duration of ADT in only 26% of patients with XRT
	Tilki et al. (42) Chicago Prostate Cancer Center, USA and Martini-Klinik Prostate Cancer Center, Germany (accrual 1992–2013)	639 patients with Gleason 9–10 treated with RP ± adjuvant RT ± ADT or XRT + BT + ADT (median 6 months)	OM, PCM	5-year PCM: 21.89% (RP), 3.93% (RP + XRT), 9.83% MaxRP, 27.04% RP + ADT vs. 5-year PCM: 2.22% (MaxRT)	Surgery and RT comparison cohorts at geographically different centers
	Reichard et al. (43) MD Anderson (accrual 2004–2013); comparison with Matched SEER Cohort	304 patients with NCCN high-risk or very-high-risk treated with RP or XRT + ADT	BF, DM, OM, LF	5-year OM RP = 4.3% RT + ADT = 1.5% HR NS	Limited patient number to assess OM or PCM endpoints; only 3.9% of RP patients received adjuvant RT, no PCM reported

*BF, biochemical failure; BT, brachytherapy; DM, distant metastases; Gy, gray; HR, hazard ratio; HRQoL, health-related quality of life; LDR, low-dose-rate; MaxRP, RP followed by adjuvant radiation within 1 year; MaxRT, XRT + brachytherapy ± ADT; OM, overall mortality; NS, not significant; PCM, prostate cancer-specific mortality; PSA, prostate-specific antigen; XRT, external beam radiation therapy*.

### Limited Ability to Investigate Mortality Endpoints With Limited-Institution Observational Data

Despite the lack of RCTs that provide data regarding high-risk disease, multiple institutions have published retrospective data comparing RP and RT outcomes. Selected studies are presented in Table 1, with a more comprehensive list in Supplementary Table 4. The largest of these are retrospective studies published by Memorial Sloan Kettering, Mayo Clinic/Fox Chase, and Cleveland Clinic (39–41). The Memorial Sloan Kettering Study described outcomes for cT1c–T3b PCa who underwent either RP with pelvic lymphadenectomy or RT (without coverage of pelvic lymph nodes) to a dose of at least 81 Gy. At a median follow-up time of 5 years, the study reported a 7.8% difference in 8-year metastatic progression in the high-risk subset favoring RP. The hazard ratio (HR) for PCM in RP vs. RT is 0.32 [95% confidence interval (CI), 0.13–0.80; *P* = 0.015] in favor of surgery after adjusting for preoperative Kattan nomogram, age, and treatment year. Adjusted HRs for the high-risk subset were not published. The treatment of high-risk patients in this study was additionally limited by the lack of long-term ADT and possibly the lack of pelvic lymph node irradiation, as has been discussed (2). There is some continuing equipoise regarding the latter issue. The relative benefit of pelvic lymph node irradiation in intermediate- to high-risk PCa is currently undergoing prospective evaluation in RTOG 0924, although the majority of clinical trials publishing outcomes for combination XRT and ADT for high-risk PCa included pelvic lymph node irradiation (7–9, 12).

Large cohorts focusing more on a high-risk group were reported by Mayo Clinic/Fox Chase (40) and Cleveland Clinic (41). The former group focused on RP vs. XRT, with the latter additionally comparing patients who received low-dose-rate (LDR) BT. The Mayo Clinic/Fox Chase group published outcomes for 1,847 National Comprehensive Cancer Network (NCCN) high-risk patients, treated with RP or XRT. Pelvic lymph node coverage was included in the radiation portal. The study principally reported a 10-year cancer-specific survival rate of 92, 92, and 88% after RP, XRT + ADT, and XRT alone, respectively (*P* = 0.06). After adjusting for case mix, there were no significant differences in systemic progression (HR, 0.78; 95% CI, 0.51–1.18; P = 0.23) or PCM (HR, 1.14; 95% CI, 0.68–1.91; *P* = 0.61) between patients who received XRT + ADT and patients who underwent RP. The risk of OM, however, was greater after XRT + ADT than after RP (HR, 1.60; 95% CI, 1.25–2.05; *P* = 0.0002). The study is strengthened by follow-up >10 years for the patients receiving RP and 6 years for patients receiving RT, as well as a median duration of ADT of 22.8 months. It is limited, though, by the low radiation doses used (72 Gy). The Cleveland Clinic cohort published cancer-specific survival outcomes of 2,557 patients with NCCN high-risk PCa treated with XRT ± ADT, LDR ± ADT, or RP ± XRT (41). The PCM at 5 and 10 years, respectively, was 5.3 and 11.2% for XRT, 3.2 and 3.6% for LDR BT, and 2.8 and 6.8% for RP (*P* = 0.0004). Although radiation dose utilized was notably higher than that of the Mayo/Fox Chase Study, with patients receiving at least 78 Gy, the utilization rate of long-course ADT in high-risk patients was low. Only 26% of patients receiving RT had an ADT duration >6 months. This low rate limits the interpretation of the HR from the study in favor of RP when comparing RP vs. XRT.

There have been more recently published single-institution studies demonstrating improved compliance with dose-escalated RT and long-course ADT, though the numbers of patients included have been demonstrably lower (43, 46). A study by Washington University reported outcomes for 62 propensity-score-matched pairs of patients with NCCN high-risk PCa receiving RP or XRT (46). Although not achieving uniform compliance, the study states that 80.6% of the patients receiving XRT received 2 years of total ADT. The median XRT dose was 75.6 Gy. Although PCM was not reported, 5-year rates of metastasis for RP and RT were 33 and 8.9%, respectively (*P* = 0.003), with no difference in overall survival detected. The more recent study was published by MD Anderson, describing a cohort of 304 patients with NCCN high or very high-risk PCa treated from 2004 to 2013 with RP or XRT + ADT (43). The XRT + ADT group included 73 patients, though 100% received ADT with a median duration of 22 months, and all but one patient received ≥75.6 Gy. At 83 months median follow-up, there was no difference in local recurrence (HR, 2.7; 95% CI, 1.0–7.9; P = 0.06), distant metastasis failure (HR, 2.5; 95% CI, 0.8–7.8; *P* = 0.1), or OM (HR, 1.35; 95 CI, 0.4–4.8; *P* = 0.6) between patients undergoing RP vs. RT + ADT, with definition of HR in this study favoring RT at higher values. Although both of these studies demonstrate improved compliance with modern XRT + ADT standard of care compared with previous single-institutional studies, the patient sample size limits statistical power to detect differences in PCM or OM.

Although large single or limited-institutional studies are available comparing RP vs. RT outcomes, the shift that only happened relatively recently to modern ADT and radiation dose regimens continues to limit interpretation of the most extensive studies. Newer single-institutional series will likely provide a more relevant comparison of optimized RT/ADT vs. RP with less confounding as data mature.

### Population-Based Databases

With the proliferation of cancer registries, a multitude of PCa outcome studies have been published utilizing large databases to examine late-term OM or PCM (47). Studies using databases with limited reporting of ADT and RT dose compliance include those utilizing PCOS [Surveillance, Epidemiology, and End Results (SEER)], PcBaSe, and other early organized databases. These databases demonstrate a somewhat limited ability to account for adequacy of combined modality therapy in the setting of high-risk treatment due to difficulty accounting for dose-escalation as well as 1.5 months or greater of ADT in the context of XRT/ADT definitive management (Supplementary Table 5) (48–51). National Cancer Database (NCDB) studies typically allow for reporting of whether a patient received ADT during or after therapy. The duration of treatment, however, is not reported. NCDB studies are also limited by no report of PCM (52–54). SEER-Medicare studies, on the other hand, have been able to report the median number of days of ADT in some instances, with assessment of both OM and PCM possible (55–57). Virtually all databases have limited difficulty to provide full details regarding the RT plan, including consideration of dose and pelvic nodal treatment. Several representative studies drawn from these databases are shown in Table 2 and Supplementary Table 4. The vast majority of population-based studies point to RT relying solely on external beam without BT as associated with worse OM (49, 51, 52, 56, 57) and PCM (48–51, 55–57) than RP. Ability to eliminate residual confounding, most notably to ensure both adequate RT dose and ADT duration, in any of these studies is limited.

**Table 2:** Selected population-based database studies comparing survival endpoints for prostatectomy vs. radiotherapy.

**References**	**Database used/accrual period**	**Cohort described**	**Key results**	**Missing variables of study/limitations of database used**
Hoffman et al. (49)	PCOS/SEER (1994–2010)	1,655, including 437 high-risk (PSA > 10 or Gleason ≥ 8) treated with RP or XRT	High-risk results: RP was associated with statistically significant advantages for OM: HR 0.65 (95% CI 0.48–0.87), and PCM: HR: 0.36 (95% CI 0.20–0.64)	ADT duration RT dose RT modality/plan details Small sample size
Sooriakumaran et al. (50)	PcBaSe Sweden (1996–2010)	32,846 including 7649 modified NCCN high-risk	HR for PCM favors RP over RT: HR = 1.50 (95% CI 1.19–1.88)	ADT use/duration RT dose RT modality/plan details
Ennis et al. (52)	NCDB (2004–2013)	Clinically localized, NCCN high-risk who received RP or XRT + ADT or XRT + BT ± ADT	No difference in OM between RP and XRT + BT, XRT/ADT associated with higher mortality than RP (HR, 1.53; 95% CI, 1.22–1.92).	ADT use/duration RT dose RT modality/plan details PCM
Jang et al. (56)	SEER-Medicare (1992–2009)	T3-T4N0M0 or T3-T4N1M0, age ≥65 treated with RP/adjuvant XRT or XRT/ADT	10-year PCM and OM favored men who underwent RP + XRT over men who underwent XRT + ADT	RT dose; RT modality/plan details; lack of specific information regarding biochemical/clinical recurrence; lack of patient-reported outcomes; data for non-Medicare beneficiaries <65 years
Muralidhar et al. (54)	NCDB and SEER (2004–2012 for NCDB and SEER)	cT1-T3N0M0, Gleason 9–10, PSA 0–40 ng/ml treated with XRT + BT or RP + ART	NCDB: No difference in 5-year OM between RP + ART vs. XRT + BT (HR 1.10, 95% CI 0.95–1.27) SEER: No difference in 5-year PCM (HR 1.22, 95% CI 0.88–1.71)	Limitations as above for SEER and NCDB studies

*ADT, androgen deprivation therapy; ART, adjuvant radiation therapy; BT, brachytherapy; HR, hazard ratio; OM, overall mortality; NCCN, National Comprehensive Cancer Network; NCDB, National Cancer Database; PCM, prostate cancer-specific mortality; PSA, prostate-specific antigen; SEER, Surveillance, Epidemiology, and End Results; XRT, external beam radiation therapy*.

### Early Array of Meta-Analyses

To cope with the difficulties presented by the lack of randomized data in the setting of high-risk PCa treated with RT vs. RP, many investigators have sought to pool the above study types to perform meta-analyses comparing outcomes of RT or RP with respect to OM and PCM. Although BCR is commonly used as a metric of clinical relapse in retrospective or observational studies of PCa, there have been arguments against whether this is a clinically meaningful endpoint. For instance, the definition of biochemical recurrence differs depending on whether patients receive upfront surgery or radiation, with the AUA definition used in the former instance and the ASTRO or Phoenix criteria most often used in the latter (58–60). Additionally, surgical data suggest that at 5 years following BCR, ~10% of men will have developed clinical progression, and ~5% will have experienced PCM, with no association on multivariate analysis between time to BCR and risk of systemic progression or PCM (40). The vast majority of meta-analyses have thus focused on OM and PCM, two measures which are difficult for individual observational studies to reliably assess because of an often large sample size and follow-up required (27–30, 32, 61, 62). The most well-known of these meta-analyses utilized pooled results from >90,000 patients for OM and PCM estimates of HRs of RT-based outcomes relative to RP in the setting of all clinically localized PCa (Table 3). The study reported that patients treated with RT had a statistically significant higher risk of death (OM, HR, 1.63; 95% CI, 1.54–1.73; PCM, HR, 2.08; 95% CI, 1.76–2.47) (30). These findings were robust to subgroup and sensitivity analysis, as well as covariates tested, including PCa risk group, RT modality, follow-up duration, study accrual period, or geographic region of the study. The authors even detected a survival benefit in favor of RP even in the setting of low-risk disease, an association that was not detected in the UK ProtecT study that was published the same year (3). The authors of this meta-analysis later commented that this result for low-risk patients in the meta-analysis was potentially caused by a statistical anomaly referred to as the Will Rogers phenomenon and, perhaps more importantly, the strong possibility of residual confounding (29, 38, 63, 64).

**Table 3:** Selected meta-analyses comparing prostate cancer-specific mortality and overall mortality between radical prostatectomy and radiation therapy.

**References**	**Study description**	**Results**	**Notable limitations**
Wallis et al. (30)	Meta-analysis of 19 studies of low to moderate risk of bias (Newcastle-Ottawa used for assessment), up to 118,830 pooled patients	Worse OM (aHR = 1.63) and PCM (aHR = 2.08) with RT compared with RP	Residual confounding, limited quality control regarding adequacy of ADT, RT dose in included studies
Roach et al. (29)	Meta-analysis of 14 studies. Stratified studies by use of “reliability score” incorporating comorbidity adjustment, ADT quality, and study size	10-year OM and PCM favored RP over RT, by 10 and 4%, respectively. Higher “reliability” associated with differences of 5.5 and 1%, respectively.	Residual confounding, use of unvalidated “reliability score” based on somewhat subjective criteria to stratify included studies

*ADT, androgen deprivation therapy; OM, overall mortality; PCM, prostate cancer-specific mortality; RP, radical prostatectomy; RT, radiation therapy*.

The findings of this meta-analysis, which has been widely cited as the definitive pooling of observational studies to date examining RP and XRT, have been scrutinized and criticized by some (65–67). Roach et al. (29) attempted to elucidate possible explanations for the magnitude of the HR in favor of surgery. While most studies reporting HRs comparing relative efficacy constructed from observational data utilized validated measures of bias such as the Newcastle-Ottawa Scale or GRADE (68, 69), there is often limited reporting of ADT use, RT modality and dose, or the use of adjuvant radiation in the setting of adverse surgical features. Roach et al. constructed a “reliability score” that incorporated a point-based system favoring studies providing full details of staging with Gleason score, T stage, and PSA. Studies were rewarded for demonstrating high compliance with recommended ADT duration for high-risk disease, whereas studies with limited reporting regarding ADT were penalized. Perhaps more controversially, extensive population-based studies across multiple institutions and those utilizing >12,000 patients were penalized, given a perceived inability to control for residual confounding. Using this somewhat controversial technique, which has been criticized by the authors of the previous meta-analysis (38), Roach et al. demonstrated that the magnitude of the HR estimator in favor of RP decreased for both OM and PCM as the deemed “reliability” of the study increased according to this metric. Although the criticism regarding the use of an unvalidated “reliability score” must be acknowledged, the study did highlight an apparent association between the estimated degree of “surgical superiority” with larger studies that incorporated limited reporting of ADT and RT compliance in the setting of high-risk disease.

The vast majority of population-based studies and meta-analyses pooling these data along with single-institution studies point to superior OM and PCM outcomes with RP over RT. Underlying these studies, however, is valid criticism surrounding the degree to which large-scale studies can account for optimized RT/ADT regimens.

## Current Questions of Interest Related to Observational Data Regarding RP vs. RT

### Interpreting Historical Results in Light of Practice-Changing Clinical Trials

Multiple practice-changing clinical trials have been reported in the past two decades that have led to changes in the NCCN recommended standard of care for high-risk PCa, should an upfront RT approach be chosen. Although the studies included heterogeneous inclusion criteria and ADT durations ranging from 4 months to lifelong treatment, multiple studies were published that provided evidence of improved disease-free survival and PCM (7–10). EORTC 22991 additionally provided evidence that disease-free survival remains improved in the setting of intermediate- to high-risk PCa with 6 months of GnRH agonist ADT at 5 years in the context of dose-escalated RT (11). There is continuing uncertainty regarding the optimal duration of ADT in the setting of high-risk disease, though multiple clinical trials have narrowed the typical range recommended by NCCN to 1.5 years or longer when ADT is used in combination with definitive XRT (12–15, 70, 71). Surveys have suggested that since the publication of trials supporting prolonged ADT in the setting of high-risk disease, compliance with longer ADT duration has increased. Notably, however, there are distinct proportions of patients up to ~50% who continue to receive short-course ADT. Concern regarding comorbidities and uncertainty in the era of dose-escalation are occasionally cited as associated with incomplete compliance (72–75). As such, investigators drawing conclusions from large observational database studies must remain cognizant that there are many reasons why current treatment patterns for high-risk disease remain heterogeneous and not necessarily consistent with level I data provided by these RCTs. Investigators who wish to make such comparisons need to take into account the ADT quality as a potential confounder, along with traditional covariates examined in modern database studies.

### Current Questions Related to BT Boost

As discussed in the Introduction, the trend of RT in clinically localized PCa, including high-risk disease, has been to explore safe dose-escalation (76–80). With increasing attention paid to both LDR and high-dose-rate (HDR) BT boost utilized in combination with XRT, observational studies providing comparison of treatment outcomes regarding RP vs. combination XRT + BT ± ADT have been recently published (42, 52–55, 81–83). This treatment regimen has sometimes been referred to as ComboRT (54) or MaxRT (42). There has been increased interest in studying treatment outcomes of this regimen since the publication of the ASCENDE-RT trial that demonstrated improved BF with the addition of I-125 LDR boost to a minimum peripheral dose of 115 Gy. This improved BF came at the cost of a higher risk of genitourinary (GU) toxicity. The ASCENDE-RT study notably included 69% of patients with high-risk disease (6) and reaffirmed earlier retrospective evidence from the Prostate Cancer Results Study Group (84).

There are no randomized data comparing XRT + BT regimens to RP. Many have sought to address this retrospectively with institutional series or multi-institutional registries (42, 81–83). Perhaps most notable among the efforts among limited institutions is a study cohort comprising 639 patients with Gleason 9–10 PCa treated either with RP with pelvic lymph node dissection in the Martini-Klinik Prostate Cancer Center in Germany (*n* = 559) or Max-RT at the Chicago Prostate Cancer Center (*n* = 80) (42). MaxRT was defined as a combination of XRT, BT, and ADT. A strength of this study was the stratification of surgical outcomes by receipt of adjuvant RT and ADT. Fifty patients received MaxRP, defined as RP followed by adjuvant XRT and ADT. The results pointed to significantly reduced PCM for Gleason 9–10 PCa with MaxRT compared to RP, with MaxRT patients receiving a median ADT duration of 6 months. Patients receiving MaxRP, however, did not demonstrate a statistically significant difference in HR for PCM or OM, with the authors computing a plausibility index for equivalence of treatment of 76.75% between the treatment arms of MaxRP and MaxRT for PCM and 77.97% for OM. One limitation is a source of bias introduced by the geographic separation between the comparator groups.

This paper, along with the Kishan study described below, has drawn the attention of others seeking to utilize large cancer databases and registries to examine the same question. Relatively few studies have been able to draw comparisons in OM (52–55) and even fewer in PCM (54, 55). One study using SEER-Medicare curiously reported a more favorable OM with XRT + BT compared with RP but not PCM (55). Another study utilizing the NCDB in a cohort with NCCN high-risk disease ≤ 65 years of age and with Charlson Comorbidity Index of 0 reported a worse OM with XRT + BT compared with RP (53), a result that was not seen in a larger cohort without age restriction (52). A recent study utilizing both NCDB for comparisons of OM and SEER for comparisons of PCM reaffirmed the apparent lack of statistically significant difference in OM or PCM when comparing MaxRT and MaxRP in patients with Gleason 9–10 disease, not observing any evidence of favorable surgical outcomes in younger patients <65 years (54). Although there is some heterogeneity among the population-based studies when different populations of high-risk disease and age are assembled, the majority of studies seem to suggest a trend toward improved PCM when XRT + BT boost is incorporated alongside ADT. Although associations of improved PCM relative to RP have been contested, there is less evidence suggesting superior surgical outcomes when BT boost is incorporated alongside XRT/ADT. These studies suggest that dose-escalation in the form of BT boost may form a crucial role in achieving superior local control when upfront RT is used.

### Multi-Institutional Registries

Many groups have sought to achieve quality control for data collection and reporting with customized multi-institutional registry studies (Table 4). Assembling large numbers of patients in a database between institutions does not by itself provide a basis for reducing the potential for residual confounding; the onus remains on participating investigators to thoughtfully survey and record classifiers that ensure quality control and facilitate necessary statistical adjustments. These registries allow improved reporting of ADT regimens and compliance with dose-escalated RT in the setting of high-risk PCa treatment while maintaining the numbers necessary to provide statistical power. Barnes-Jewish Hospital and Cleveland Clinic conducted an early such effort. They published results of 10,429 patients with clinically localized PCa treated with upfront RP, XRT, or BT regimens, including 1,234 patients with D'Amico high-risk disease (85). The authors found XRT associated with increased OM and PCM compared to RP, with BT associated with increased OM but not PCM. Propensity-matched adjusted HRs were reported. This study had the advantage of 82% of high-risk patients receiving XRT or BT receiving ADT. The study is limited by the low radiation dose used and ADT duration delivered for many patients, which are considered insufficient by current standards. A subsequent study conducted by Duke University, Chicago, and twenty-first Century Oncology focused solely on patients <75 years of age with clinically localized Gleason 8–10 disease, treated with either XRT + BT with ADT or RP (81). Patients received ADT for a median of 4.3 months, which started before BT. Their study found that RP was not associated with an increased risk of PCM compared with XRT + BT with ADT, reporting an HR of 1.8 (95% CI, 0.6–5.6).

**Table 4:** Multi-institutional registry studies.

**Author, Institutions**	**Inclusion criteria**	**Comparison**	**Findings**
Kibel: Barnes-Jewish Hospital and Cleveland Clinic (1995–2005) (85)	Clinically localized disease; general cohort of 10,429 including 1,234 D'Amico high-risk patients	XRT/ADT or BT vs. RP Note: XRT/ADT—median 74 Gy (Barnes-Jewish) or 78 Gy (Cleveland Clinic) and 82% of high-risk patients received ADT (median 6 months)	Worse OM with XRT/ADT (HR, 1.7; 95% CI, 1.3–2.3) or BT (HR, 3.1; 95% CI, 1.7–5.9) compared with RP, though no detectable difference in PCM in high-risk subset Adjusted 10-year PCM of 1.8% (RP), 2.9% (XRT), or 2.3% (BT)
Westover: 21st Century Oncology, Chicago Prostate Center, Duke University (1988–2008) (81)	Clinically localized, Gleason 8–10, age <75 657 patients included	XRT + BT vs. RP Note: XRT + BT included 45 Gy + minimum 90–108 Gy BT	No detectable difference in PCM, i.e., PCM for RP not detected as worse than CMT (HR, 1.8; 95% CI, 0.6–5.6)
Kishan: 12 tertiary centers (11 in the United States, 1 in Norway) from 2000 to 2013 (83)	Gleason 9–10, clinically localized disease	XRT + BT (MaxRT) vs. RP Note: XRT- median 74.3 Gy, XRT+BT median 91.5 Gy, pelvic nodes included in 40.7% of XRT patients	Improved OM and PCM with MaxRT compared with RP Adjusted 5-year PCM RP 12% (95% CI, 8–17%); EBRT 13% (95% CI, 8–19%); and EBRT + BT, 3% (95% CI, 1–5%) PCM HR MaxRT vs. RP−0.38 (95% CI, 0.21–0.68) OM HR MaxRT vs. RP−0.66 (95% CI, 0.46–0.96)

*ADT, androgen deprivation therapy; BT, brachytherapy; CMT, combined modality therapy; Gy, gray; MaxRT, combination external beam radiation therapy and brachytherapy ± ADT; OM, overall mortality; PCM, prostate cancer-specific mortality; RP, radical prostatectomy; RT, radiation therapy; XRT, external beam RT*.

**Table 5:** Selected clinical trials studying various therapies including systemic, surgical, and radiation interventions in high-risk prostate cancer.

**NCT ID #**	**Phase**	**Title**	**Accrual and arms**	**Treatment arms**
**Systemic therapy interventions**				
NCT03477864	1	Stereotactic body radiation therapy with REGN2810 and/or ipilimumab before surgery in treating participants with progressive advanced or oligometastatic prostate cancer	Active target enrollment: 24 three arms	Anti-PD1 monoclonal antibody (REGN2810) vs. intraprostatic ipilimumab vs. a combination of both, followed by SBRT + RP
NCT02023463	1	Enzalutamide, radiation therapy, and hormone therapy in treating patients with intermediate or high-risk prostate cancer	No longer recruiting actual enrollment: 25 one arm	Enzalutamide + LHRH agonist with goserelin or leuprolide, followed by RT and additional LHRH agonist
NCT03177460	1	Daratumumab or FMS inhibitor JNJ-40346527 before surgery in treating patients with high-risk, resectable localized or locally advanced prostate cancer	Active target enrollment: 30 two arms	Daratumumab (CD38 antagonist) vs. FMS inhibitor JNJ-40346527(CSF-1R tyrosine kinase inhibitor) followed by RP
NCT00099086	1	Docetaxel, radiation therapy, and hormone therapy in treating patients with locally advanced prostate cancer	No longer recruiting actual enrollment: 20 one arm	RT + bicalutamide and GnRH analog prior to, during, and after RT + concurrent docetaxel
NCT03821246	2	Neoadjuvant atezolizumab with or without enzalutamide in localized prostate cancer given before radical prostatectomy	Active target enrollment: 68 three arms	Atezolizumab alone vs. in combination with enzalutamide or in combination with emactuzumab, followed by RP
NCT02506114	2	Neoadjuvant PROSTVAC-VF with or without ipilimumab for prostate cancer	Active target enrollment: 75 two arms	PROSTVAC-VF (PSA-based immunization) ± ipilimumab, followed by RP
NCT02508636	2	Trial of radiotherapy with leuprolide and enzalutamide in high-risk prostate	No longer recruiting actual enrollment: 11 one arm	Definitive RT + Leuprolide + Enzalutamide
NCT02772588	2	AASUR in high-risk prostate cancer	Active target enrollment: 58 one arm	Leuprolide + Abiraterone + apalutamide + SBRT
NCT02903368	2	Neoadjuvant and adjuvant abiraterone acetate + apalutamide prostate cancer undergoing prostatectomy	No longer recruiting actual enrollment: 120 two arms, crossover	Abiraterone, leuprolide, prednisone ± apalutamide, followed by RP. Adjuvant abiraterone, apalutamide, leuprolide, prednisone vs. no adjuvant therapy.
NCT03436654	2	Multi-arm multi-modality therapy for very high-risk localized and low volume metastatic prostatic adenocarcinoma	Active target enrollment: 76 two arms	Apalutamide ± (Abiraterone and prednisone) followed by RP, pelvic lymphadenectomy, GnRH agonist/antagonist
NCT03432780	2	Radiation-hormone and docetaxel vs. radiation-hormone in patients with high-risk localized prostate cancer (QRT-SOGUG)	No longer recruiting actual enrollment: 134 two arms	RT + hormone therapy ± weekly docetaxel
NCT01385059	2	Axitinib before surgery in treating patients with high-risk prostate cancer	No longer recruiting actual enrollment: 60 two arms	Axitinib for 28 days vs. no therapy followed by RP and pelvic lymph node dissection
NCT02849990	2	A phase II neoadjuvant study of apalutamide, abiraterone acetate, prednisone, degarelix and indomethacin in men with localized prostate cancer pre-prostatectomy	No longer recruiting actual enrollment: 22 one arm	Apalutamide, abiraterone, prednisone, degarelix, indomethacin followed by RP
NCT03899987	2	Aspirin and rintatolimod with or without interferon-alpha 2b in treating patients with prostate cancer before surgery	Active target enrollment: 60 two arms	Aspirin + rintatolimod ± recombinant interferon alpha-2b followed by RP vs. RP alone
NCT02949284	2	Androgen receptor antagonist ARN-509 with or without abiraterone acetate, gonadotropin-releasing hormone analog, and prednisone in treating patients with high-risk prostate cancer undergoing surgery	Active target enrollment: 90 two arms	Apalutamide ± (abiraterone acetate, GnRH agonist, prednisone) followed by RP vs. RP alone
NCT01409200	2	Antiandrogen therapy with or without axitinib before surgery in treating patients with previously untreated prostate cancer with known or suspected lymph node metastasis	No longer recruiting actual enrollment: 73 two arms	ADT + axitinib followed by RP and pelvic lymph node dissection vs. ADT alone followed by RP and pelvic lymph node dissection
NCT01546987	3	Hormone therapy, radiation therapy, and steroid 17alpha-monooxygenase TAK-700 in treating patients with high-risk prostate cancer	No longer recruiting actual enrollment: 239 two arms	ADT + GnRH agonist + RT ± TAK-700 (steroid 17alpha-monooxygenase)
NCT03767244	3	A study of apalutamide in participants with high-risk, localized or locally advanced prostate cancer who are candidates for radical prostatectomy (PROTEUS)	Active target enrollment: 1,500 two arms	ADT + apalutamide OR placebo, followed by RP, followed by adjuvant ADT + apalutamide OR placebo
NCT00288080	3	Hormone therapy and radiation therapy or hormone therapy and radiation therapy followed by docetaxel and prednisone in treating patients with localized prostate cancer	No longer recruiting actual enrollment: 612 two arms	Androgen suppression with LHRH agonist + oral anti-androgen prior to and concurrent with RT, followed by adjuvant LHRH agonist ± docetaxel x six cycles
NCT00430183	3	Surgery with or without docetaxel and leuprolide or goserelin in treating patients with high-risk localized prostate cancer	No longer recruiting actual enrollment: 788 two arms	Docetaxel + LHRH agonist + surgery vs. surgery alone
**Surgical interventions**				
NCT00007644	3	Prostate cancer intervention vs. observation trial (PIVOT)	Results published; pending long-term results actual enrollment: 731 two arms	RP vs. observation
N/A	3	Radical prostatectomy or watchful waiting in early prostate cancer (SPCG-4)	Results published; pending long-term results actual enrollment: 695 two arms	Watchful waiting vs. RP
NCT02102477	3	Surgery vs. radiotherapy for locally advanced prostate cancer (SPCG-15)	Active target enrollment: 1,200 two arms	RP ± adjuvant or salvage RT, vs. RT with adjuvant ADT
**Radiation therapy interventions**				
NCT02830165	1	Stereotactic body radiation therapy in treating patients with high-risk prostate cancer undergoing surgery	Active target enrollment: 12 one arm	SBRT given over three fractions ~2–4 weeks prior to RP
NCT02346253	1 | 2	High-dose brachytherapy in treating patients with prostate cancer	Active target enrollment: 163 one arm	High-dose brachytherapy over two fractions + ADT
NCT00951535	2	A prospective phase II dose-escalation study using IMRT for high-risk N0 M0 prostate cancer. ICORG 08-17	No longer recruiting actual enrollment: 251	Dose-escalation study from baseline of 75.6 Gy up to a maximum of 81 Gy, depending on volume constraints
NCT01368588	3	Androgen-deprivation therapy and radiation therapy in treating patients with prostate cancer (RTOG 0924)	No longer recruiting actual enrollment: 2,592 two arms	RT to prostate and seminal vesicles alone vs. whole-pelvis RT
NCT00967863	3	Radiation therapy in treating patients receiving hormone therapy for prostate cancer (GETUG-AFU 18)	No longer recruiting actual enrollment: 500 two arms	RT to 80 Gy vs. to 70 Gy given in conjunction with ADT
NCT00667888	3	A phase III intensity radiotherapy dose-escalation for prostate cancer using hypofractionation	No longer recruiting actual enrollment: 225 two arms	RT to 75.6 Gy in 42 fractions vs. RT to 72 Gy in 30 fractions
**Other interventions**				
NCT03514927	2	High-intensity focused ultrasound in treating participants with intermediate and high-risk prostate cancer	Active target enrollment: 32 one arm	High-intensity focused ultrasound (HIFU) followed by RP

*ADT, androgen deprivation therapy; Gy, gray; IMRT, intensity-modulated radiation therapy; LHRH, Luteinizing hormone-releasing hormone; PSA, prostate-specific antigen; RP, radical prostatectomy; RT, radiation therapy; SBRT, stereotactic body radiotherapy*.

The most impressive multi-institutional registry endeavor to date was conducted by the University of California, Los Angeles, the California Endocurie Therapy Center, and Fox Chase, broadening to include 12 tertiary centers. These two studies focused on Gleason 9–10 PCa, comparing PCM, OM, and distant metastasis for patients receiving RP, XRT with ADT, or XRT + BT combined with ADT (82, 83). The more extensive publication included 1,809 patients. The authors reported high-quality ADT, including XRT and XRT + BT arms that received 89.5 and 92.4% utilization of ADT as part of the initial treatment strategy, with median durations of 21.9 and 12 months, respectively. After the inverse probability of treatment weighting adjustments, XRT + BT was associated with a significantly longer time until distant metastases (DM) and lower PCM than either XRT or RP. One potential limitation to interpreting the outcomes from the RP arm includes the relatively low utilization of adjuvant RT of 8.7% for Gleason 9–10 disease, with salvage performed in 34.1% of patients. On examination of subgroups by radiation dose, patients receiving <70 Gy had a significantly higher rate of PCM than either those receiving ≥78 Gy, though this relationship did not hold with DM. The registry did not record and control for comorbidity status, which is a limitation regarding adjustment of HRs between RT and RP. The authors speculated that the lack of this information was unlikely to bias their conclusion in favor of RT, as RT cohorts typically have more comorbidities.

These efforts, conducted by urologists and radiation oncologists alike, have contributed unique opportunities to assemble large cohorts of patients across institutions with a level of quality control regarding recommended treatment compliance, perhaps second only to prospectively organized studies or RCTs. It is likely that these efforts will continue to answer questions too detailed for standard cancer registries, yet impossible to address with currently available RCT data.

### Addition of Systemic Therapies; Limited Data Available From Randomized Trials

There has been much interest in both past and current clinical trials to explore the addition of chemotherapy to long-term ADT and dose-escalated RT, with currently available prospective randomized trials demonstrating limited follow-up (23–26). Available data from GETUG 12, RTOG 0521, and the non-metastatic subgroup of STAMPEDE point to an improved relapse-free survival associated with the use of docetaxel in patients treated with XRT + ADT (24, 25, 86), with recently updated data from RTOG 0521 demonstrating an additional improvement in DM (24). Data are still maturing from the majority of available clinical trials, and the decision to use chemotherapy is currently individualized based on patient disease characteristics. There are limited data, especially regarding the interplay between MaxRT incorporating BT and systemic therapy strategies. More extensive recent systematic review and discussion of currently available prospective trials, additionally including consideration of second-generation ADT and adjuvant treatments following surgery, are provided elsewhere (26).

## Current Prospective Trials of Interest

There are multiple active clinical trials currently underway investigating various promising therapeutic interventions for high-risk PCa. The majority of these clinical trials are early-phase (phase I or II). The principal role of these studies is to investigate the role of additional systemic treatment modalities or evaluate the most effective timing of systemic therapy in relation to definitive local treatment modalities such as surgery or radiation. Furthermore, with the surge in interest in immunotherapies for cancer as a whole, high-risk PCa has been seen as a potential area for the integration of further immune treatments into the current standard of care. For example, trial NCT03477864 investigates the safety of injecting intravenous anti-PD1 monoclonal antibody and intraprostatic ipilimumab (either alone or in combination with each other) in the setting of high-risk PCa, to be followed by both SBRT and RP as definitive local modalities. In addition, a phase II trial (NCT02506114) is evaluating the efficacy of a PSA-based form of immunization, with or without additional immunotherapy with ipilimumab, prior to definitive local treatment with RP for high-risk PCa. This is not to say that traditional chemotherapeutic regimens have been overlooked, however; the QRT-SOGUG phase II trial (NCT03432780), of which a preliminary report of results was published in abstract format in 2016, is investigating the safety and efficacy of administering weekly docetaxel concurrently with standard dosing of RT and ADT (87). In addition, there is interest in adapting hormone therapy regimens to incorporate the newest generation of drugs such as apalutamide and abiraterone; the phase II trial NCT02949284 is a trial that is examining the feasibility of performing nerve-sparing RP in the setting of apalutamide given either alone or in combination with abiraterone and prednisone.

There have been relatively fewer phase III trials investigating the role of surgical intervention in high-risk PCa. These include the PIVOT trial, which most recently reported results in 2017 with a median of 12.7 years of follow-up; the PIVOT trial randomly assigned 731 individuals with a diagnosis of localized PCa to observation or RP, and found that the RP arm did not have significantly lower OM or PCM compared to the observation arm (88). Notably, there was a trend toward significance for these metrics in the higher-risk populations–namely, those with a PSA value of >10 and a Gleason score of 7 or higher. In contrast, the SPCG-4 trial, which randomized 695 men with localized PCa to watchful waiting or RP, did demonstrate a benefit to surgery, with a number needed to treat to prevent one death of eight (89). Nevertheless, in the high-risk group of patients in this trial, there was no significant difference in OM, PCM, and risk of metastases, as of the most recent results in 2014 with over 23 years of follow-up. The next generation of trials is found in the phase III SPCG-15 trial, which is currently active and recruiting with a target enrollment of 1,200 patients (45). This trial enrolls patients with locally advanced PCa and randomizes them to either standard of care with radiation and ADT vs. RP (including extended pelvic lymph node dissection) with adjuvant or salvage radiation and hormone therapy if necessary; the primary endpoint is cause-specific survival.

Also, multiple trials are actively investigating variations upon the currently accepted dose, fractionation scheme, and method of delivery of RT for locally advanced high-risk PCa. Phase I trials such as NCT02830165 investigate the safety of adding of hypofractionated stereotactic RT given prior to RP, with the hypothesis that providing patients with two forms of local therapy may aid in increased disease control as well as prompt an immune response in high-risk disease (90). The phase I/II trial NCT02346253, which is not limited to high-risk patients but includes patients up to T3 and a Gleason score of 10, seeks to answer whether HDR-BT delivered over two fractions, in conjunction with ADT and luteinizing hormone-releasing hormone (LHRH) agonist therapy, is safe and efficacious (as assessed by the rates of genitourinary toxicity, PSA nadir, and rates of freedom from biochemical failure). How radiation fields should be defined–that is, in terms of whole-pelvis RT vs. RT to the prostate and seminal vesicles alone–is under active investigation as well in the trial NCT01368588 (RTOG 0924). With an accrual of over 2,500 patients, it is powered to answer the question of overall survival differential between these two radiation field setups in patients with a moderate or high risk of recurrence. Dose-escalation continues to be actively investigated as well. For example, NCT00967863 (GETUG-AFU 18) examines the impact of dose-escalation to 80 Gy (vs. a standard of 70 Gy) in high-risk PCa patients in a phase III randomized setting; as of November 2015, there was no increased toxicity noted acutely or at 1-year follow-up, but the results for biochemical and clinical control are still pending at this time (91). Other local treatment options have continued to remain of interest. The phase II single-arm trial NCT03514927 seeks to determine whether high-intensity focused ultrasound (HIFU) can be used in conjunction with RP to impact the percent of viable cancer tissue noted on the surgical pathology specimen. Overall, the general thrust of the new emerging data and clinical trials appears to be in adding various systemic therapies, particularly in new hormonal treatments and incorporation of immunotherapies, with some studies also looking at the role of surgery and modifying current radiation techniques and dosages.

## Conclusions

So far, RCTs assessing OM and PCM between RP and RT are limited concerning representation of high-risk, clinically localized disease. Most observational studies and meta-analyses have not historically supported oncologic equivalence between the two modalities. Issues of selection bias, inadequate use of ADT/radiation dose, and residual confounding remain as difficulties in interpreting available retrospective data. Trends have demonstrated more recent curation of multi-institutional registries and databases. These have allowed assessment of outcomes for patients receiving treatment showing improved compliance with modern evidence-based RT/ADT and RP/adjuvant RT regimens. High-dose RT incorporating BT boost has demonstrated evidence of improved PCM. With more recent efforts, estimates of differences in PCM between RT- and RP-based approaches have diminished. There is still a relative lack of prospective randomized trials being organized to provide a comparison between RP and RT strategies, with the majority of trials exploring new hormonal therapies, immunotherapies, and in general augmentation of existing strategies. Likely, retrospective data will still be a significant resource in answering questions regarding the interplay of RP- and RT-based modalities for high-risk PCa.

## Author Contributions

BG and RD provided study conception. BG and VC provided initial manuscript preparation, with BG, VC, and RD providing critical revisions and preparation of the final manuscript. RD provided study oversight.

### Conflict of Interest

The authors declare that the research was conducted in the absence of any commercial or financial relationships that could be construed as a potential conflict of interest.
