# Supplementary material for: Combined Modality Therapies for High-Risk Prostate Cancer: Narrative Review of Current Understanding and New Directions
Source: Front Oncol. 2019 Nov 29;9:1273. doi: 10.3389/fonc.2019.01273 (PMC6896415; doi:10.3389/fonc.2019.01273)
Supplement: Supplementary file 1 [file Data_Sheet_1.pdf]

Search Strings Utilized for Respective Databases
Pubmed
(("Prostatic Neoplasms"[MeSH]) OR prostate carcinoma OR prostate cancer OR (“Prostatic Neoplasms, Castration-Resistant” [MeSH]) OR prostate tumor OR prostate tumour)<br><br>AND<br><br>(Gleason 8 OR Gleason 9 OR Gleason 10 OR high risk OR high-risk)<br><br>AND<br><br>((“prostatectomy”[MeSH]) OR prostatectomy OR resection OR surgery)<br><br>AND<br>(("Drug Therapy"[MeSH]) OR ("Combined Modality Therapy "[MeSH]) OR ("Radiotherapy"[MeSH]) OR (external beam radiation therapy) OR brachytherapy OR xrt OR ebrt OR (conformal radiotherapy) OR (IMRT) OR (VMAT) OR (Intensity-modulated) OR (high-energy RT) OR (image guided RT) OR (androgen deprivation therapy) OR (ADT) OR (hormonal therapy) OR (“Therapeutics” [MeSH]) OR (therapeutics) OR (“Chemoradiotherapy” [MeSH]) OR chemoradiotherapy OR (systemic therapy))<br><br>And<br><br>((“Cohort Studies” [MeSH]) OR Cohort analysis OR (“longitudinal studies”[MeSH]) OR longitudinal study OR (“prospective studies”[MeSH]) OR prospective study OR (“Case Control Studies” [MeSH]) OR Case Control Study OR Hospital based case control study OR population based case control study OR (“retrospective studies”[MeSH]) OR retrospective study) OR ((“Outcome Assessment (Health Care)”[MeSH]) OR (“Treatment Outcome”[MeSH]) OR (“Recurrence”[MeSH]) OR Cancer recurrence OR Cancer regression OR Cancer relapse OR Disease Duration OR Disease Exacerbation OR (“Prognosis”[MeSH]) OR Prognosis OR Recurrent Disease OR Relapse OR Remission OR Tumor Recurrence OR tumor regression OR survival OR cancer survival OR disease free survival OR overall survival OR (“Survival”[MeSH]) OR Survival OR (“Mortality”[MeSH]) OR Mortality OR (“Disease-Free Survival”[MeSH]) OR Disease-free survival OR (“Survival Rate”[MeSH]) OR Survival Rate OR (“Survival Analysis”[MeSH]) OR survival analysis OR (“Treatment Outcome”[MeSH]) OR treatment outcome OR (“Treatment Failure”[MeSH]) OR treatment failure OR (“Disease Progression”[MeSH]) OR disease progression OR (“Cause of Death”[MeSH]) OR cause of death OR (“Kaplan-Meier Estimate”[MeSH]) OR Kaplan-Meier Estimate OR (“Proportional Hazards Models”[MeSH]) OR proportional hazards models OR (“Propensity Score” [MeSH]) OR Propensity Score)
Scopus
TITLE-ABS-KEY (Prostatic W/2 Neoplasms) OR TITLE-ABS-KEY (prostate W/2 carcinoma) OR TITLE-ABS-KEY (prostate W/2 cancer) OR TITLE-ABS-KEY (Prostatic W/2 Neoplasms, Castration-Resistant) OR TITLE-ABS-KEY(prostate W/2 tumor)<br><br>AND<br><br>TITLE-ABS-KEY ({gleason 8}) OR TITLE-ABS-KEY ({gleason 9}) OR TITLE-ABS-KEY ({gleason 10}) OR TITLE-ABS-KEY (high AND risk) OR TITLE-ABS-KEY ({high-risk})<br><br>AND<br><br>TITLE-ABS-KEY (prostatectomy) OR TITLE-ABS-KEY (resection) OR TITLE-ABS-KEY (surgery)<br><br>AND<br><br>(TITLE-ABS-KEY (Drug W/2 Therapy) OR TITLE-ABS-KEY (Combined W/2 Modality W/2 Therapy) OR TITLE-ABS-

KEY (Radiotherapy) OR TITLE-ABS-KEY ({external beam radiation therapy}) OR TITLE-ABS-KEY (brachytherapy) OR TITLE-ABS-KEY (XRT) OR TITLE-ABS-KEY (EBRT) OR TITLE-ABS-KEY ({conformal AND radiotherapy}) OR TITLE-ABS-KEY (IMRT) OR TITLE-ABS-KEY (VMAT) OR TITLE-ABS-KEY (Intensity-modulated) OR TITLE-ABS-KEY ({Intensity Modulated}) OR TITLE-ABS-KEY(high-energy AND RT) OR TITLE-ABS-KEY (image guided AND RT) OR TITLE-ABS-KEY (androgen AND deprivation AND therapy) OR TITLE-ABS-KEY(ADT) OR TITLE-ABS-KEY(hormonal AND therapy) OR TITLE-ABS-KEY (therapeutics) OR TITLE-ABS-KEY (chemoradiotherapy) OR TITLE-ABS-KEY (systemic W/2 therapy))

AND

((TITLE-ABS-KEY (Cohort AND Studies) OR TITLE-ABS-KEY (Cohort AND Analysis) OR TITLE-ABS-KEY (Longitudinal AND Studies) OR TITLE-ABS-KEY (Prospective AND Studies) OR TITLE-ABS-KEY (Case W/2 Control W/2 Studies) OR TITLE-ABS-KEY (Hospital AND Based AND Case AND Control AND Study) OR TITLE-ABS-KEY (Population AND Based AND Case AND Control AND Study) OR TITLE-ABS-KEY (Retrospective AND Studies) OR TITLE-ABS-KEY (Retrospective AND Studies))

OR

(TITLE-ABS-KEY (Outcome AND Assessment) OR TITLE-ABS-KEY (Treatment AND Outcome) OR TITLE-ABS-KEY (Cancer AND Recurrence) OR TITLE-ABS-KEY (Cancer AND Regression) OR TITLE-ABS-KEY (Cancer AND Relapse) OR TITLE-ABS-KEY (Disease AND Duration) OR TITLE-ABS-KEY (Disease AND Exacerbation) OR TITLE-ABS-KEY (Prognosis) OR TITLE-ABS-KEY (Recurrent AND Disease) OR TITLE-ABS-KEY (Relapse) OR TITLE-ABS-KEY (Remission) OR TITLE-ABS-KEY (Tumor AND Recurrence) OR TITLE-ABS-KEY (tumor AND regression) OR TITLE-ABS-KEY (survival) OR TITLE-ABS-KEY (cancer AND survival) OR TITLE-ABS-KEY (disease AND free AND survival) OR TITLE-ABS-KEY (overall AND survival) OR TITLE-ABS-KEY (Mortality) OR TITLE-ABS-KEY (Disease-Free AND Survival) OR TITLE-ABS-KEY (Survival AND Rate) OR TITLE-ABS-KEY (Survival AND Analysis) OR TITLE-ABS-KEY (Treatment AND Outcome) OR TITLE-ABS-KEY (Treatment AND Failure) OR TITLE-ABS-KEY (Disease AND Progression) TITLE-ABS-KEY (Cause AND Death) OR TITLE-ABS-KEY (Kaplan-Meier AND Estimate) OR TITLE-ABS-KEY (Proportional AND Hazards AND Models) OR TITLE-ABS-KEY (Propensity AND Score)))

Cochrane Database Clinical Trials

([mh "Prostatic Neoplasms"] OR prostate carcinoma OR prostate cancer OR [mh “Prostatic Neoplasms, Castration-Resistant”] OR prostate tumor OR prostate tumour)

AND

(Gleason 8 OR Gleason 9 OR Gleason 10 OR high risk OR high-risk)

AND

([mh “prostatectomy”] OR prostatectomy OR resection OR surgery)

AND

([mh “Drug Therapy”] OR [mh "Combined Modality Therapy"] OR [mh “Radiotherapy”] OR (external beam radiation therapy) OR brachytherapy OR xrt OR ebrt OR (conformal radiotherapy) OR (IMRT) OR (VMAT) OR (Intensity-modulated) OR (high-energy RT) OR (image guided RT) OR (androgen deprivation therapy) OR (ADT) OR (hormonal therapy) OR [mh “Therapeutics”] OR (therapeutics) OR [mh “Chemoradiotherapy”] OR chemoradiotherapy OR (systemic therapy))

And

(([mh “Cohort Studies”] OR Cohort analysis OR [mh “longitudinal studies”] OR longitudinal study OR [mh “prospective studies”] OR prospective study OR [mh “Case-Control Studies”] OR Case Control Study OR Hospital based case control study OR population based case control study OR [mh “retrospective studies”] OR retrospective study) OR ([mh “Outcome Assessment (Health Care)”] OR [mh “Treatment Outcome”] OR [mh

“Recurrence”] OR Cancer recurrence OR Cancer regression OR Cancer relapse OR Disease Duration OR Disease Exacerbation OR [mh “Prognosis”] OR Prognosis OR Recurrent Disease OR Relapse OR Remission OR Tumor Recurrence OR tumor regression OR survival OR cancer survival OR disease free survival OR overall survival OR [mh “Survival”] OR Survival OR [mh “Mortality”] OR Mortality OR [mh “Disease-Free Survival”] OR Disease-free survival OR [mh “Survival Rate”] OR Survival Rate OR [mh “Survival Analysis”] OR survival analysis OR [mh “Treatment Outcome”] OR treatment outcome OR [mh “Treatment Failure”] OR treatment failure OR [mh “Disease Progression”] OR disease progression OR [mh “Cause of Death”] OR cause of death OR [mh “Kaplan-Meier Estimate”] OR Kaplan-Meier Estimate OR [mh “Proportional Hazards Models”] OR proportional hazards models OR [mh “Propensity Score”] OR Propensity Score))

Search results were compiled after which duplicate publications were removed, supplemented with manual curation of resulting publication database. After the elimination of duplicate references, 3739 references were screened. Screening within the search results for full-text articles targeted publications with abstracts subject to peer-review published within the past decade, including randomized controlled, cohort, population, or registry studies, and case-control studies describing outcomes for at least 100 patients. The publication window (1/1/2009 – 8/7/2019) was established given trends in dose escalation for definitive XRT, paradigm changes in ADT use, and surgical advances.

Supplementary Table 1: Search Terms Incorporated for Respective Databases

Supplementary Table 2: Definitions of High Risk Included

Definition	High-risk criteria
D’Amico et al.	Stage T2c or greater and either PSA level > 20 ng/ml or Gleason 8-10
RTOG 99-02 and 05-21	PSA 20-100 ng/ml, Gleason score ≥ 7 and any T stage; stage T2 or greater, PSA < 100 ng/ml and Gleason score 8-10
NCCN high or very high risk	PSA level > 20 mg/L or Gleason score 8-10 or stage T3 or greater, with very high risk corresponding to T3b-T4, primary Gleason Score =5, or >4 cores with Gleason 8-10
American Urologic Association	PSA ≥ 20 ng/mL or Gleason score ≥ 8 or clinical stage T ≥ T2c
European Association of Urology	PSA ≥ 20 ng/mL or Gleason score ≥ 8 or clinical stage T ≥ T3a
Radiation Therapy Oncology Group	Gleason Score = 7 with Clinical Stage T3 or N1; Gleason Score ≥ 8 and Clinical Stage T1-2, with very high risk including Clinical Stage T3b-T4

Supplementary Table 3: Search Terms for Clinical Trials

Terms and Synonyms Searched:

Terms / Synonyms	Search Results*	Entire Database**
**Prostate Cancer Stage III**	64 studies	275 studies
stage III prostate cancer	61 studies	270 studies
Stage III Prostate Carcinoma	1 studies	2 studies
**Prostate Cancer Stage**	2 studies	36 studies
stage prostate cancer	--	29 studies
**Stage III**	80 studies	4,131 studies
stage 3	--	165 studies
stage three	--	4 studies
third stage	--	42 studies
**Cancer Stage**	3 studies	298 studies
Cancer Staging	--	21 studies
Neoplasm Staging	--	13 studies
Tumor Staging	--	3 studies
**Prostate Cancer**	82 studies	4,405 studies
Prostatic Neoplasm	81 studies	3,834 studies
prostate carcinoma	8 studies	217 studies
Carcinoma of the Prostate	1 studies	17 studies
Cancer of prostate	--	31 studies
cancer of the prostate	--	68 studies
CARCINOMA OF PROSTATE	--	3 studies
carcinoma prostate	--	4 studies
neoplasia prostate	--	1 studies
Neoplasm of prostate	--	5 studies
neoplasm of the prostate	--	1 studies
Prostate neoplasia	--	2 studies
Prostate Neoplasms	--	89 studies
prostate tumors	--	15 studies
prostatic cancer	--	54 studies
prostatic carcinoma	--	16 studies
Tumor of prostate	--	1 studies
Tumor of the Prostate	--	2 studies
**III**	82 studies	11,007 studies
Third	--	638 studies
**Stage**	82 studies	61,196 studies
Phase	44 studies	55,068 studies
Phased	--	22 studies
**Cancer**	82 studies	69,449 studies
Neoplasm	82 studies	60,712 studies
Tumor	9 studies	15,477 studies
Malignancy	--	2,963 studies
Neoplasia	--	589 studies
Neoplastic Disease	--	19 studies
neoplastic syndrome	--	587 studies
Oncology	--	1,108 studies
**Prostate**	82 studies	5,052 studies
Prostatic	81 studies	4,364 studies
Prostata	--	3 studies

-- No studies found

* Number of studies in the search results containing the term or synonym

** Number of studies in the entire database containing the term or synonym

Supplemental Table 4: Comparative Effectiveness Methods in High-Risk Prostate Cancer

Study Type:	Representative Studies	Findings	Key Limitations
Randomized Trials	Lennernäs (2015)[1]<br>Akakura (2006)[2]	No difference in PCM between RP and RT-based approaches	Limited sample size, lack of statistical power
Single or Limited Multi-Institution Studies	Reichard (2019)[3]<br>Caño-Velasco (2019)[4]<br>Tilki (2018)[5]<br>Koo (2018)[6]<br>Markovina (2018)[7]<br>Ciezki (2017)[8]<br>Taguchi (2015)[9]<br>Yamamoto (2014)[10]<br>Merino (2013) [11]<br>Boorjian (2011)[12]<br>Zelefsky (2010)[13]	Widely different conclusions regarding PCM, with OM most often demonstrating improvement associated with RP when reported	Limited sample size, limited follow-up, often focus on biochemical outcomes instead of OM/PCM. Compliance with dose-escalated RT and ADT only recently demonstrating improvement; Residual confounding
Population-based Database	Jayadevappa (2019)[14]<br>Muralidhar (2019)[15]<br>Berg (2018)[16]<br>Ennis (2018)[17]<br>Gu (2018)[18]<br>Jang (2018)[19]<br>Sooriakumaran (2014)[20]<br>Hoffman (2013)[21]<br>Abdollah (2012)[22]	Widely different cohorts assembled to represent subsets of high-risk patients; Most often OM and PCM measures favor RP over RT even after propensity matching	Residual confounding, inadequate reporting of RT dose, plan, ADT quality and duration. RT patients typically older with higher comorbidity index with limited ability to adjust
Multi-Institutional Registries	Kishan (2017-2018)[23,24]<br>Kibel (2012)[25]<br>Westover (2012)[26]	No detectable improvement in PCM with RP over RT<br><br>Improved investigation of MaxRT strategies (EBRT+BT +/- ADT) with suggestion of improved PCM with MaxRT over RP, and not compared with RP followed by adjuvant RT	Limited sample size; practice variation between institutions with variable adherence to ADT practices;<br><br>Time and effort required to assemble a large cohort tailored to specific questions manually
Systematic Review / Meta-Analyses	Serrell (2018)[27]<br>Wallis (2016)[28]<br>Roach (2015)[29]<br>Lei (2015)[30]<br>Petrelli (2014)[31]	Improved OM and PCM with RP over RT for clinically localized prostate cancer	Residual confounding; limited reporting of component studies regarding ADT quality, RT dose; limited ability to adjust for this with validated techniques in meta-analysis[29]<br><br>Limited reporting of MaxRT strategies

Supplemental Table 5: Available Population Databases used for Comparison of Prostate Cancer Outcomes

Prostate Cancer Databases	Selected Variables of Interest Included	Notably Missing Variables of Interest	Representative Studies
SEER	Prostate cancer incidence, initial treatments, disease characteristics, pathologic characteristics, PCM, cause of death	ADT use / duration<br>RT dose<br>RT modality/plan details<br>Comorbidity<br>Treatment complications<br>Patient-reported outcomes	Abdollah[22]<br>Gu[18]<br>Muralidhar[15]
PCOS	Sexual function, urinary, bowel function; health-related quality of life; disease characteristic; comorbidities, PCM, cause of death	ADT duration<br>RT dose<br>RT modality/plan details<br>Small sample size	Hoffman[21]
PCBaSe	Inpatient and outpatient care, detailed follow-up, prescription medications, patterns of care	ADT use / duration<br>RT dose<br>RT modality/plan details	Sooriakumaran[20]
NCDB	Charlson comorbidity index; detailed information regarding disease characteristics	ADT use / duration<br>RT dose<br>RT modality/plan details<br>PCM	Ennis[17]<br>Berg[16]<br>Muralidhar[15]
SEER-Medicare	Detailed disease characteristics; comorbidities; treatment complications; data regarding treatment type and adjuvant/salvage therapies	RT dose;<br>RT modality/plan details;<br>Lack of specific information regarding biochemical/clinical recurrence; lack of patient-reported outcomes; Data for non-Medicare beneficiaries < 65 years	Jayadevappa[14]<br>Jang[19]

References:

1. Lennernäs B, Majumder K, Damber J-E, et al. Radical prostatectomy versus high-dose irradiation in localized/locally advanced prostate cancer: A Swedish multicenter randomized trial with patient-reported outcomes. *Acta Oncologica*. 2015;54(6):875-881. doi:10.3109/0284186X.2014.974827

2. Akakura K, Suzuki H, Ichikawa T, et al. A Randomized Trial Comparing Radical Prostatectomy Plus Endocrine Therapy versus External Beam Radiotherapy Plus Endocrine Therapy for Locally Advanced Prostate Cancer: Results at Median Follow-up of 102 Months. *Japanese Journal of Clinical Oncology*. 2006;36(12):789-793. doi:10.1093/jjco/hyl115

3. Reichard CA, Hoffman KE, Tang C, et al. Radical Prostatectomy or Radiotherapy for High and Very High Risk Prostate Cancer: A Multidisciplinary Clinic Experience of Patients Eligible for Either Treatment. *BJU International*. April 2019. doi:10.1111/bju.14780

4. Caño-Velasco J, Herranz-Amo F, Barbas-Bernardos G, et al. Differences in overall survival and cancer-specific survival in high-risk prostate cancer patients according to the primary treatment. *Actas Urológicas Españolas (English Edition)*. 2019;43(2):91-98. doi:10.1016/j.acuroe.2018.06.009

5. Tilki D, Chen M-H, Wu J, et al. Surgery vs Radiotherapy in the Management of Biopsy Gleason Score 9-10 Prostate Cancer and the Risk of Mortality. *JAMA Oncology*. November 2018. doi:10.1001/jamaoncol.2018.4836

6. Koo KC, Cho JS, Bang WJ, et al. Cancer-Specific Mortality Among Korean Men with Localized or Locally Advanced Prostate Cancer Treated with Radical Prostatectomy Versus Radiotherapy: A Multi-Center Study Using Propensity Scoring and Competing Risk Regression Analyses. *Cancer Research and Treatment*. 2018;50(1):129-137. doi:10.4143/crt.2017.004

7. Markovina S, Meeks MW, Badiyan S, et al. Superior metastasis-free survival for patients with high-risk prostate cancer treated with definitive radiation therapy compared to radical prostatectomy: A propensity score-matched analysis. *Advances in Radiation Oncology*. 2018;3(2):190-196. doi:10.1016/j.adro.2017.12.001

8. Ciezki JP, Weller M, Reddy CA, et al. A Comparison Between Low-Dose-Rate Brachytherapy With or Without Androgen Deprivation, External Beam Radiation Therapy With or Without Androgen Deprivation, and Radical Prostatectomy With or Without Adjuvant or Salvage Radiation Therapy for High-Risk Prostate Cancer. *International Journal of Radiation Oncology*Biology*Physics*. 2017;97(5):962-975. doi:10.1016/j.ijrobp.2016.12.014

9. Taguchi S, Fukuhara H, Shiraishi K, et al. Radical Prostatectomy versus External Beam Radiotherapy for cT1-4N0M0 Prostate Cancer: Comparison of Patient Outcomes Including Mortality. Sung S-Y, ed. *PLOS ONE*. 2015;10(10):e0141123. doi:10.1371/journal.pone.0141123

10. Yamamoto S, Kawakami S, Yonese J, et al. Long-term oncological outcome in men with T3 prostate cancer: radical prostatectomy versus external-beam radiation therapy at a single institution. *International Journal of Clinical Oncology*. 2014;19(6):1085-1091. doi:10.1007/s10147-013-0654-2

11. Merino T, San Francisco IF, Rojas PA, Bettoli P, Zúñiga Á, Besa P. Intensity-modulated radiotherapy versus radical prostatectomy in patients with localized prostate cancer: long-term follow-up. *BMC Cancer*. 2013;13(1). doi:10.1186/1471-2407-13-530

12. Boorjian SA, Karnes RJ, Viterbo R, et al. Long-term survival after radical prostatectomy versus external-beam radiotherapy for patients with high-risk prostate cancer. *Cancer*. 2011;117(13):2883-2891. doi:10.1002/cncr.25900

13. Zelefsky MJ, Eastham JA, Cronin AM, et al. Metastasis After Radical Prostatectomy or External Beam Radiotherapy for Patients With Clinically Localized Prostate Cancer: A Comparison of Clinical Cohorts Adjusted for Case Mix. *Journal of Clinical Oncology*. 2010;28(9):1508-1513. doi:10.1200/JCO.2009.22.2265

14. Jayadevappa R, Lee DI, Chhatre S, Guzzo TJ, Malkowicz SB. Comparative effectiveness of treatments for high-risk prostate cancer patients. *Urologic Oncology: Seminars and Original Investigations*. July 2019. doi:10.1016/j.urolonc.2019.06.005

15. Muralidhar V, Mahal BA, Butler S, et al. Combined external beam radiation therapy and brachytherapy versus radical prostatectomy with adjuvant radiation therapy for Gleason 9-10 prostate cancer. *Journal of Urology*. May 2019. doi:10.1097/JU.0000000000000352

16. Berg S, Cole AP, Krimphove MJ, et al. Comparative Effectiveness of Radical Prostatectomy Versus External Beam Radiation Therapy Plus Brachytherapy in Patients with High-risk Localized Prostate Cancer. *European Urology*. 2019;75(4):552-555. doi:10.1016/j.eururo.2018.10.032

17. Ennis RD, Hu L, Ryemon SN, Lin J, Mazumdar M. Brachytherapy-Based Radiotherapy and Radical Prostatectomy Are Associated With Similar Survival in High-Risk Localized Prostate Cancer. *Journal of Clinical Oncology*. 2018;36(12):1192-1198. doi:10.1200/JCO.2017.75.9134

18. Gu X, Gao X, Cui M, et al. Survival outcomes of radical prostatectomy and external beam radiotherapy in clinically localized high-risk prostate cancer: a population-based, propensity score matched study. *CMAR*. 2018;Volume 10:1061-1067. doi:10.2147/CMAR.S157442

19. Jang TL, Patel N, Faiena I, et al. Comparative effectiveness of radical prostatectomy with adjuvant radiotherapy versus radiotherapy plus androgen deprivation therapy for men with advanced prostate cancer. *Cancer*. 2018;124(20):4010-4022. doi:10.1002/cncr.31726

20. Sooriakumaran P, Nyberg T, Akre O, et al. Comparative effectiveness of radical prostatectomy and radiotherapy in prostate cancer: observational study of mortality outcomes. *BMJ*. 2014;348(feb26 6):g1502-g1502. doi:10.1136/bmj.g1502

21. Hoffman RM, Koyama T, Fan K-H, et al. Mortality After Radical Prostatectomy or External Beam Radiotherapy for Localized Prostate Cancer. *JNCI Journal of the National Cancer Institute*. 2013;105(10):711-718. doi:10.1093/jnci/djt059

22. Abdollah F, Schmitges J, Sun M, et al. Comparison of mortality outcomes after radical prostatectomy versus radiotherapy in patients with localized prostate cancer: A population-based analysis: Prostate cancer treatment. *International Journal of Urology*. 2012;19(9):836-844. doi:10.1111/j.1442-2042.2012.03052.x

23. Kishan AU, Shaikh T, Wang P-C, et al. Clinical Outcomes for Patients with Gleason Score 9–10 Prostate Adenocarcinoma Treated With Radiotherapy or Radical Prostatectomy: A Multi-institutional Comparative Analysis. *European Urology*. 2017;71(5):766-773. doi:10.1016/j.eururo.2016.06.046

24. Kishan AU, Cook RR, Ciezki JP, et al. Radical Prostatectomy, External Beam Radiotherapy, or External Beam Radiotherapy With Brachytherapy Boost and Disease Progression and Mortality in Patients With Gleason Score 9-10 Prostate Cancer. *JAMA*. 2018;319(9):896. doi:10.1001/jama.2018.0587

25. Kibel AS, Ciezki JP, Klein EA, et al. Survival Among Men With Clinically Localized Prostate Cancer Treated With Radical Prostatectomy or Radiation Therapy in the Prostate Specific Antigen Era. *Journal of Urology*. 2012;187(4):1259-1265. doi:10.1016/j.juro.2011.11.084

26. Westover K, Chen M-H, Moul J, et al. Radical prostatectomy vs radiation therapy and androgen-suppression therapy in high-risk prostate cancer: *RP VS CMT FOR ADVANCED PROSTATE CANCER*. *BJU International*. 2012;110(8):1116-1121. doi:10.1111/j.1464-410X.2012.11012.x

27. Serrell EC, Pitts D, Hayn M, Beaule L, Hansen MH, Sammon JD. Review of the comparative effectiveness of radical prostatectomy, radiation therapy, or expectant management of localized prostate cancer in registry data. *Urologic Oncology: Seminars and Original Investigations*. 2018;36(4):183-192. doi:10.1016/j.urolonc.2017.10.003

28. Wallis CJD, Saskin R, Choo R, et al. Surgery Versus Radiotherapy for Clinically-localized Prostate Cancer: A Systematic Review and Meta-analysis. *European Urology*. 2016;70(1):21-30. doi:10.1016/j.eururo.2015.11.010

29. Roach M, Ceron Lizarraga TL, Lazar AA. Radical Prostatectomy Versus Radiation and Androgen Deprivation Therapy for Clinically Localized Prostate Cancer: How Good Is the Evidence? *International Journal of Radiation Oncology*Biology*Physics*. 2015;93(5):1064-1070. doi:10.1016/j.ijrobp.2015.08.005

30. Lei JH, Liu LR, Wei Q, et al. Systematic Review and Meta-analysis of the Survival Outcomes of First-line Treatment Options in High-risk Prostate Cancer. *Scientific Reports*. 2015;5(1). doi:10.1038/srep07713

31. Petrelli F, Vavassori I, Coinu A, Borgonovo K, Sarti E, Barni S. Radical Prostatectomy or Radiotherapy in High-Risk Prostate Cancer: A Systematic Review and Metaanalysis. *Clinical Genitourinary Cancer*. 2014;12(4):215-224. doi:10.1016/j.clgc.2014.01.010
